# Seasonal Differences in Migration Routes and Stopover Use of Greater Sand Plovers Between Mongolia and the Beibu Gulf Revealed by GPS Tracking

**DOI:** 10.1002/ece3.73914

**Published:** 2026-06-29

**Authors:** Lei Xu, Zhiying Cheng, Tao Meng, Gang Yang, Aiwu Jiang

**Affiliations:** ^1^ Guangxi Key Laboratory of Forest Ecology and Conservation, School of Forestry Guangxi University Nanning Guangxi China; ^2^ Guangxi Forest Inventory and Planning Institute Nanning Guangxi China

**Keywords:** Beibu Gul, migration, movement ecology, shorebird, stopover

## Abstract

Seasonal migration strategies can vary with life history demands and environmental context, yet such contrasts remain poorly documented for many shorebirds along the East Asian–Australasian Flyway (EAAF). We used high‐resolution GPS‐GSM tracking of 11 adult Greater Sand Plovers ( *Charadrius leschenaultii* ) tagged in the Beibu Gulf, China, during 2021–2022 to quantify and compare their spring (northbound; 12 tracks) and autumn (southbound; 8 tracks) migrations between nonbreeding areas in the northern South China Sea and breeding grounds in Mongolia. Spring movements followed predominantly inland corridors and were relatively direct, with journeys of approximately 3300 km completed in about 2 weeks. During spring migration, birds spent just over half of the total migration period at generally short but more frequent stopovers. In contrast, autumn movements occurred mainly along coastal pathways and covered around 4100 km over nearly 4 weeks. Across seasons, tracked individuals converged on the Beibu Gulf, which functioned both as an important stopover bottleneck and as a nonbreeding endpoint for part of the tracked population. These tracking data indicate seasonal differences in route use, stopover allocation, and habitat use, with spring movements showing a higher travel‐to‐stopover ratio and autumn movements involving a greater proportion of time at stopovers, suggesting greater refueling investment. Given the limited sample size and number of complete annual tracks, these inferences should be treated as preliminary. Nevertheless, the results highlight the potential importance of conserving both inland wetlands and coastal intertidal habitats along the EAAF.

## Graphical abstract

GPS‐GSM tracking of Greater Sand Plovers along the East Asian‐Australasian Flyway revealed seasonal differences in migration routes and stopover allocation between the northern Beibu Gulf and Mongolia. Spring northward migration was associated with a higher travel‐to‐stopover ratio, whereas autumn southward migration involved a greater proportion of time at stopovers and greater use of coastal regions. These results highlight the northern Beibu Gulf as a dual‐function region, serving both as a wintering area and a migratory stopover region for this species.

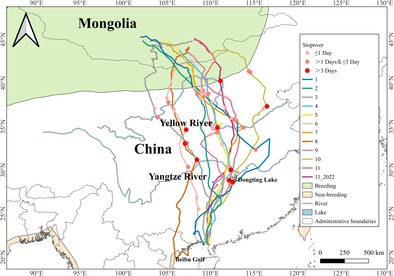

## 1 Introduction

Migration represents a fundamental life‐history strategy that enables birds, particularly waterbirds, to exploit seasonally shifting resources across vast spatial and temporal scales (Newton 2007; Byholm et al. 2022). For many species, migration is not a fixed or instinctive routine but a flexible process continually optimized under ecological constraints such as weather, food availability, and predation risk (Alerstam 2011). Waterbirds, which depend on dynamic wetland habitats, exemplify this flexibility: they adjust their routes, timing, and stopover use in response to hydrological and climatic variability. These adaptive movements allow individuals to balance competing pressures of time, energy, and safety. For example, birds often prioritize time minimization in spring to reach breeding sites early, whereas autumn movements may place greater emphasis on energy conservation and preparation for the nonbreeding season (Herbert et al. 2022; Hedenström and Hedh 2024). However, how these seasonal trade‐offs are expressed in routes, timing, and stopover use depends strongly on the broader flyway context.

This context is particularly important along the East Asian–Australasian Flyway (EAAF), one of the world's most species‐rich yet most threatened flyways (MacKinnon et al. 2012). The EAAF supports more than 50 million migratory waterbirds, including shorebirds, from over 280 species. It links breeding grounds in Arctic and Central Asia with nonbreeding sites across Southeast Asia and Australasia (Bamford et al. 2008; Mundkur and Langendoen 2022). However, the EAAF has experienced rapid and extensive habitat loss, especially in intertidal and inland wetlands, driven by agricultural expansion, tidal flat reclamation for aquaculture and coastal development, and urbanization. Consequently, these changes have fragmented essential stopover networks required for refueling and rest, leading to steep population declines across multiple taxa (Studds et al. 2017; Mcduffie et al. 2022; Zhang et al. 2025). Against this backdrop of accelerating environmental change, effective conservation planning increasingly requires better description of how individual migrants move and use key sites across the flyway, especially where tracking data remain sparse.

Recent technological advances, particularly GPS loggers and satellite transmitters, have revolutionized avian migration research by revealing fine‐scale routes, stopover dynamics, and behavioral adjustments that were previously undetectable by traditional banding alone (Flack et al. 2022). These high‐resolution movement data now allow researchers to delineate complete migration routes, pinpoint critical stopover sites, and measure individual‐level variation in timing and behavior at fine spatial and temporal scales (Chan et al. 2022; An et al. 2024; Peng et al. 2024; He et al. 2025). Earlier tracking studies in the EAAF and connected West Pacific Flyway first revealed broadscale migration routes, long‐distance connectivity, and breeding or nonbreeding distributions of migratory shorebirds, including Eastern Curlews, Bar‐tailed Godwits, and other long‐distance waders (Driscoll and Ueta 2002; Gill et al. 2009; Battley et al. 2012). More recent high‐resolution tracking studies have increasingly resolved annual routines, migratory connectivity, site fidelity, stopover function, and environmental drivers of movement at finer spatial and temporal scales (Wang et al. 2024; Zhao et al. 2026). Nevertheless, such detailed tracking evidence remains taxonomically and geographically uneven across EAAF shorebirds. In particular, relatively few studies have examined how seasonal movements are structured around key stopover regions or how individuals diverge in their post‐arrival decisions at these sites.

The Greater Sand Plover ( *Charadrius leschenaultii* ) is a long‐distance migratory shorebird that breeds across Central and East Asia and winters widely from Southeast Asia to Australasia (BirdLife International 2019). Within the EAAF, it is commonly recorded on sandy beaches, tidal flats, estuaries, and other shallow coastal habitats during the nonbreeding season (Wiersma et al. 2020). Yet accumulating evidence suggests that its seasonal movements may be more variable than this traditional view implies. Previous tracking and ringing studies of Greater Sand Plovers, including light‐level geolocator work from north‐western Australia, indicate that many Greater Sand Plovers use predominantly inland routes during northward migration (Minton et al. 2011). In contrast, extensive coastal waterbird surveys during autumn consistently report large numbers of Greater Sand Plovers at intertidal and nearshore habitats along the EAAF (Liao et al. 2024). Together, these findings raise the possibility of seasonal differences in route use and habitat association, but such differences remain insufficiently described using high‐resolution tracking data within the same migratory system.

The aim of this study was to characterize spring and autumn migration routes, stopover sites, and seasonal differences in movement metrics of GPS‐tracked Greater Sand Plovers within the EAAF. We define a migration segment as the observed portion of a seasonal journey between two major seasonal areas or stopover regions. Using high‐resolution GPS‐GSM tracking data, we described all observed migration segments and restricted direct seasonal comparisons to the Mongolia–northern Beibu Gulf segment, where spring and autumn observations were most comparable. Specifically, we asked: (1) Which spring and autumn routes and stopover sites were used by tracked Greater Sand Plovers? (2) What seasonal differences in route use, route directness, timing, and stopover allocation were evident in the migration segments available for comparison? (3) Are these differences broadly consistent with previously proposed contrasting seasonal optimization pressures? We also aimed to identify key stopover areas and habitats of potential conservation importance within the Greater Sand Plover's migratory flyway. Such information is needed to guide flyway‐scale conservation planning for this species, particularly because habitat loss and uneven protection of inland and coastal wetlands continue to threaten migratory shorebirds along the EAAF.

## 2 Methods

### 2.1 Study Area

**FIGURE 1:**
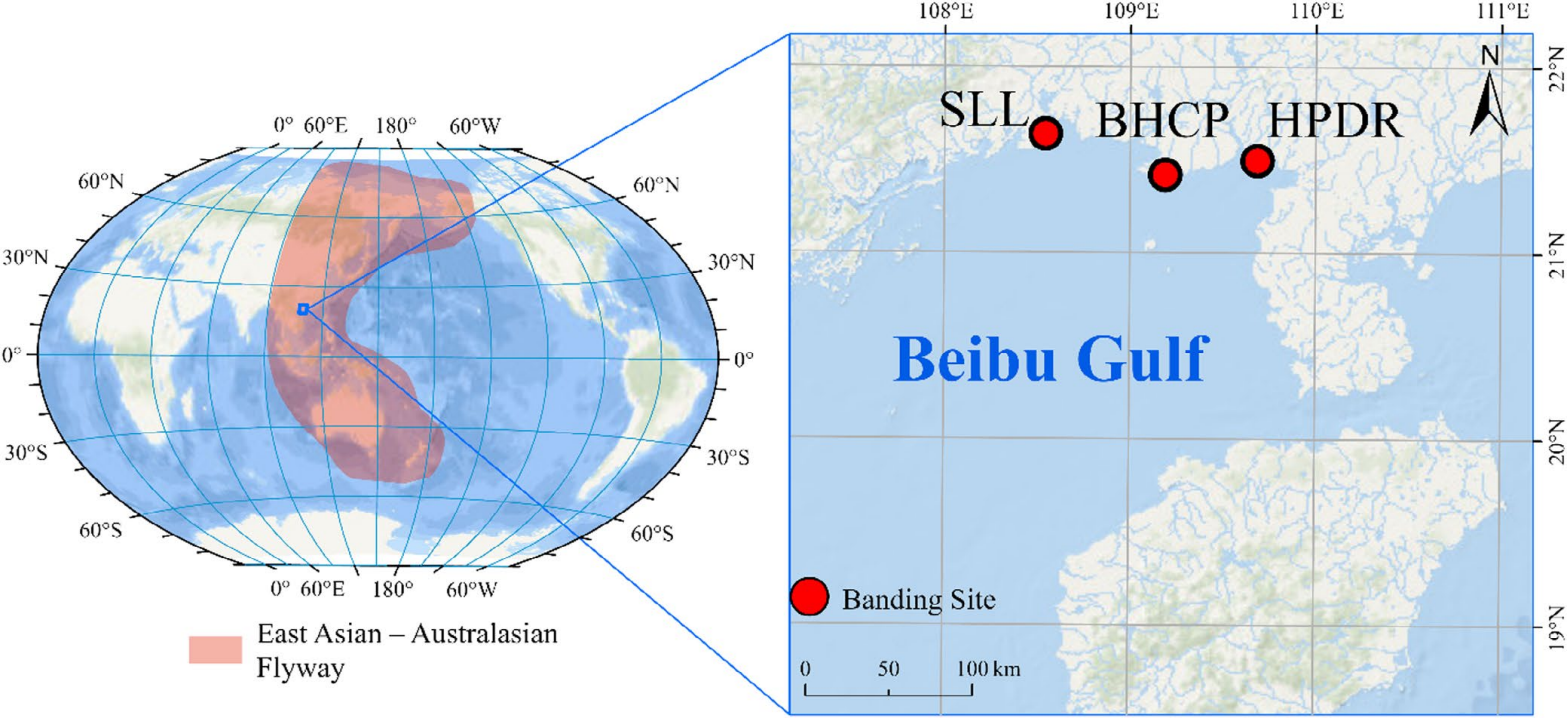
Location of Guangxi study area in the northern Beibu Gulf coast, China, showing the main capture sites of GPS‐GSM‐tracked Greater Sand Plovers along the EAAF in 2021. Capture sites were Guangxi Shaluoliao Village (SLL), Guangxi Beihai Coastal National Wetland Park (BHCP), Guangxi Hepu Dugong National Nature Reserve (HPDR).

This study was conducted during 2021–2022 in the Guangxi coastal region of southern China, along the northern Beibu Gulf, which is an important part of the EAAF (Figure 1). This area encompasses extensive mudflats, mangroves, and coastal wetlands that provide essential foraging and roosting habitats for migratory shorebirds (Wen et al. 2024). It functions both as a major nonbreeding ground and as an important stopover site for numerous migratory waterbirds (Tang et al. 2023).

### 2.2 Bird Tracking

Adult Greater Sand Plovers were captured in the Guangxi coastal region of southern China during 2021 using mist nets at coastal roosting or foraging sites during high tide periods (Figure 1). After capture, birds were removed promptly from the nets, placed in cloth bags, and processed individually. Each bird was aged, measured, weighed, fitted with a 4‐g solar‐powered GPS‐GSM transmitter (Hunan Global Messenger Technology Co. Ltd., Hunan, China; length × width × height = 22.5 × 18 × 9 mm), and released at the capture site. Transmitters were attached using leg‐loop harnesses made of flexible silicone tubing. All tagged individuals were identified as adults at the time of deployment based on morphology and plumage characteristics (Prater et al. 2007). Sex was not determined because molecular sexing was not conducted and reliable sex‐specific plumage characters were not recorded consistently for all individuals. The combined transmitter and harness mass was approximately 4.5 g, equivalent to 4.46% ± 0.16% of individual body mass at deployment, below the commonly used 5% guideline (Barron et al. 2010; Geen et al. 2019). Devices were programmed to record a GPS fix every 1–6 h. Because the analysis was based on original GPS fixes, no interpolation or state‐space regularization was applied before route reconstruction and calculation of migration metrics. Original timestamps were retained, and movement metrics were calculated from consecutive observed GPS locations.

GPS fixes were transmitted remotely through the GSM network and downloaded from the transmitter platform. In total, 18 individuals were captured and tagged in 2021 (Table S1). Unless otherwise stated, spring and autumn migrations refer to movements recorded during the annual cycle following deployment. Prior to analysis, we screened the dataset and retained individuals that yielded identifiable spring and/or autumn migration segments. We define a migration segment as the observed portion of a seasonal journey between two major seasonal areas or stopover regions. Because all transmitters were deployed in the northern Beibu Gulf, northward movements from nonbreeding areas south of the Beibu Gulf could not be fully reconstructed for all individuals. Therefore, route descriptions included all identifiable observed migration segments, whereas direct seasonal comparisons were restricted to the Mongolia–northern Beibu Gulf segment, where spring and autumn observations were most comparable. One individual (ID 11) provided tracking data for two spring and two autumn migrations. To avoid pseudoreplication in seasonal comparisons, only one annual record from this individual was included in the main paired analysis; the additional annual record was retained for route description only. Because all transmitters were deployed in the Beibu Gulf, some birds may already have commenced spring northward migration from nonbreeding areas farther south and were captured while using the Beibu Gulf as a stopover site en route to the breeding grounds. Consequently, northward movements before arrival in the Beibu Gulf could not be reconstructed for those individuals. We therefore distinguished between (i) complete seasonal journeys that were fully observed within the tracked extent and (ii) migration segments suitable for route description. Direct seasonal comparisons were restricted to the Mongolia–Beibu Gulf segment, for which observations were most comparable across seasons.

Location accuracy classes were provided by the transmitter platform based on the estimated horizontal positioning error of each GPS fix. The platform classified fixes into five levels: A (< 5 m), B (5–10 m), C (10–20 m), D (20–100 m), and E (> 100 m). To ensure reliability, only fixes with accuracy Classes A–C (estimated error < 20 m) were retained for analysis. From 2021 to 2022, this yielded 49,818 location data points from the 11 tracked individuals (Table 1).

**TABLE 1:** Overview of GPS‐GSM tracking deployments on Greater Sand Plovers captured in Guangxi, China, 2021–2022.

Bird ID	Banding site	Longitude/latitude	Tracking period	Number of GPS fixes used in analysis
1	Guangxi Beihai Coastal National Wetland Park, China	109.686° E, 21.496° N	2021/4/19 to 2021/5/15	2484
2			2021/3/28 to 2021/5/5	1819
4			2021/4/19 to 2021/9/12	2709
10			2021/4/19 to 2022/3/26	8452
3	Guangxi Hepu Dugong National Nature Reserve, China	109.189° E, 21.419° N	2021/3/31 to 2021/7/11	3821
5			2021/4/19 to 2021/9/23	4277
7			2021/3/29 to 2021/9/14	4208
9			2021/4/19 to 2021/12/2	2727
11			2021/3/31 to 2022/10/24	13,713
6	Guangxi Shaluoliao Village, China	108.543° E, 21.636° N	2021/3/17 to 2021/8/31	2060
8			2021/3/17 to 2021/10/26	3548

### 2.3 Routes and Seasonal Differences in Migration

Two movement states were identified from the tracking data: stationary periods and flight periods. Stationary periods were defined as consecutive GPS fixes with no detectable displacement, whereas flight periods were characterized by speeds > 40 km/h (Minton et al. 2011). Using the classified data and the geometric functions in QGIS (version 3.40.4), we constructed the migration routes of the tracked Greater Sand Plovers.

We also calculated migration‐segment distance, migration straightness, movement‐phase travel rate, observed migration‐segment duration, the number of stopovers, the proportion of time spent at stopovers, mean stopover duration, and the travel‐to‐stopover ratio (see Table 2 for definitions) (Nilsson et al. 2013; Clements et al. 2025). We defined the broad breeding and nonbreeding ranges of the Greater Sand Plover according to BirdLife International/IUCN range polygons to provide an external and standardized reference independent of our tracking data (BirdLife International 2019). These range maps were used only to distinguish migration periods from breeding and nonbreeding periods, not to define fine‐scale habitat use. The breeding locations of the tracked individuals in Mongolia were consistent with the broad breeding range. The start and end of a migration event were defined as the times when an individual departed from or arrived at the breeding or nonbreeding grounds. Stopover sites were identified using rigorously defined spatiotemporal criteria. A stopover event was registered when both of the following conditions were satisfied (Clements et al. 2025): (1) Six or more consecutive points within a 30 km radius, or the individual remained in an area for at least 6 h; (2) the location was outside of a defined breeding or nonbreeding area. The Greater Sand Plovers in this study were captured in the Beibu Gulf, an area that serves as both a stopover site and a nonbreeding area; for some individuals, it also functioned as a wintering endpoint. Therefore, some individuals may have commenced spring migration before tracking began. Consequently, the spring migration data from the Beibu Gulf to the breeding grounds in Mongolia might be incomplete. To ensure the robustness and comparability of our analysis, we therefore limited our comparison of seasonal migration strategies to the Mongolia–China segment of the migration routes, rather than aggregating incomplete tracks for direct seasonal comparison.

**TABLE 2:** Definitions and units of migration metrics of Greater Sand Plovers along the EAAF.

Metric	Definition
Migration‐segment distance (km)	Cumulative distance between consecutive observed GPS locations from the start to the end of the observed migration segment
Observed migration‐segment duration (days)	Time elapsed between departure from the origin area and arrival at the destination area within the observed migration segment. Departure was defined as the first sustained directional movement away from the origin area, and arrival was defined as the first settlement within the destination area. Premigratory fattening was not included
Total stopover duration (days)	Total time spent at all identified stopover sites during the observed migration segment
Movement duration	Observed migration‐segment duration minus total stopover duration
Movement‐phase travel rate (km/day)	Migration‐segment distance divided by movement duration; this metric excludes stopover time and should not be interpreted as overall migration speed
Route straightness	Great circle distance between the start and end of the observed migration segment divided by migration‐segment distance
Number of stopovers	Number of stopover events identified during the observed migration segment
Mean stopover duration	Total stopover duration divided by the number of stopovers
Proportion of time at stopovers	Total stopover duration divided by observed migration‐segment duration
Travel‐to‐stopover ratio	Migration‐segment distance divided by total stopover duration

Seasonal comparisons of migration metrics were restricted to individuals with paired spring and autumn records. To avoid pseudoreplication, each bird contributed only one record to the primary analysis; for the individual tracked over two annual cycles, only one annual record was retained. To reduce redundancy among derived migration metrics, primary seasonal comparisons focused on six biologically interpretable metrics representing route structure, migration timing, stopover use, and travel relative to stopover investment: migration‐segment distance, observed migration‐segment duration, route straightness, number of stopovers, proportion of time at stopovers, and travel‐to‐stopover ratio. Other derived metrics were retained as descriptive summaries in the Supporting Information but were not included in the primary set of statistical tests. For each metric, paired seasonal differences were first assessed for normality using the Shapiro–Wilk test. Metrics whose paired differences did not strongly deviate from normality were compared using paired *t*‐tests, whereas metrics with nonnormal paired differences were compared using Wilcoxon signed‐rank tests. To control the family‐wise error rate across multiple migration metrics, *p*‐values were adjusted using the Holm‐Bonferroni method. All statistical analyses were conducted in R version 4.2.2.

## 3 Results

### 3.1 Migration Date and Route

Spring migration occurred from April to May (Table S2), with individuals departing from coastal wetlands in the Beibu Gulf and migrating northwards to breeding grounds in Mongolia via various flyways. The spring migration took an average of 13.53 ± 4.24 days. Departures were concentrated around late April, with the peak arrival occurring in early May.

**FIGURE 2:**
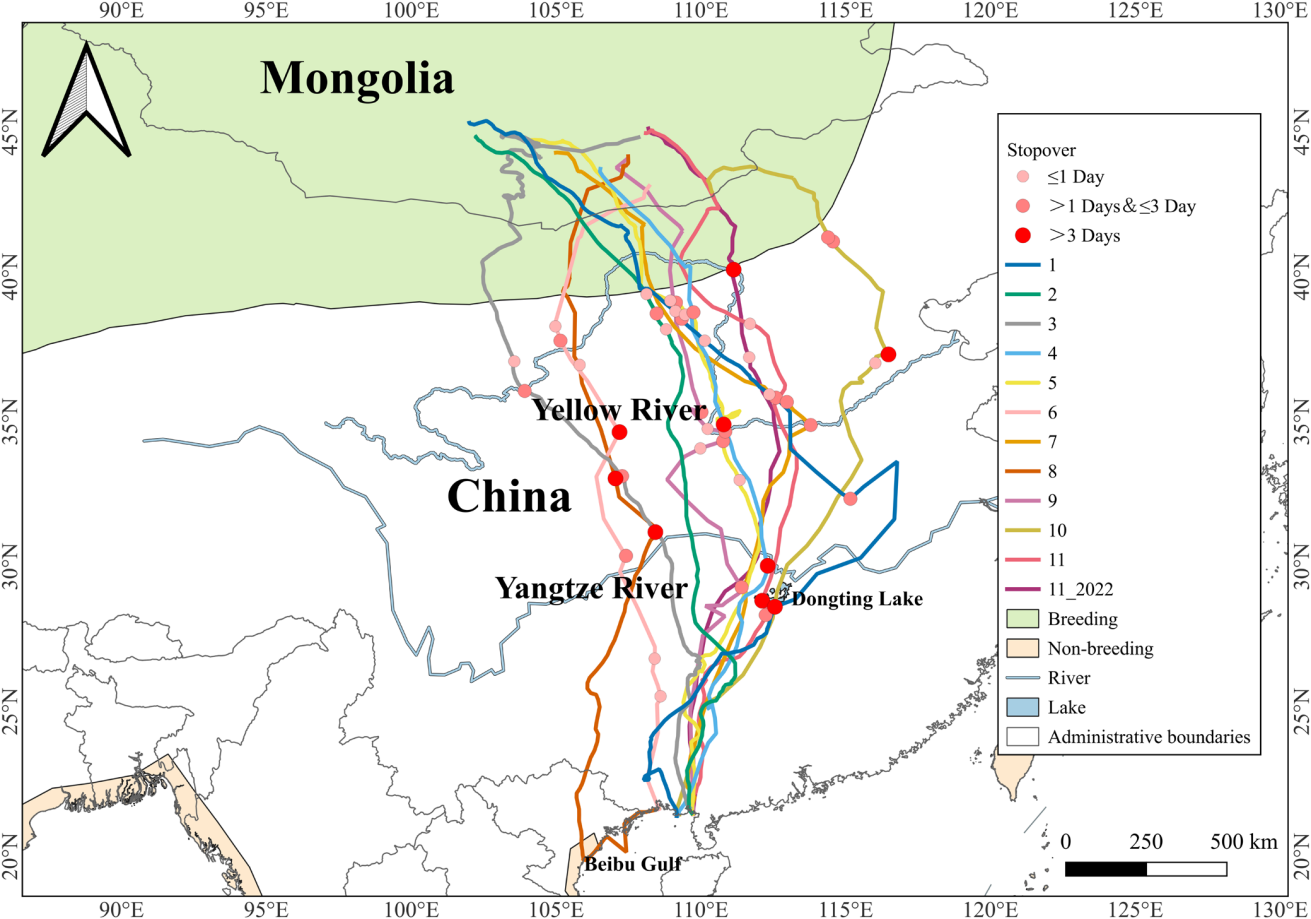
Spring northward migration routes and stopover sites of GPS‐tracked Greater Sand Plovers from the northern Beibu Gulf to Mongolia. Lines represent individual migration routes, and circles indicate stopover sites, with darker red symbols representing longer stopover duration. The shaded green and orange areas represent the breeding and nonbreeding ranges, respectively, and major rivers and lakes are shown to aid geographic interpretation.

We recorded 12 spring migration routes from 11 tracked individuals during 2021–2022, because one individual contributed spring migration data from 2 years (Figure 2). Overall, migration paths were largely concentrated in inland regions of central and eastern China. Some individuals (*n* = 3) followed a westernmost route during the early stage of migration, passing through Guizhou–Chongqing–Gansu/Ningxia–Inner Mongolia before reaching the breeding grounds. The majority of individuals (*n* = 8) migrated along a central flyway traversing South China–Central China–North China, whereas a single individual followed an easterly route via Hunan–Shandong–Hebei.

**FIGURE 3:**
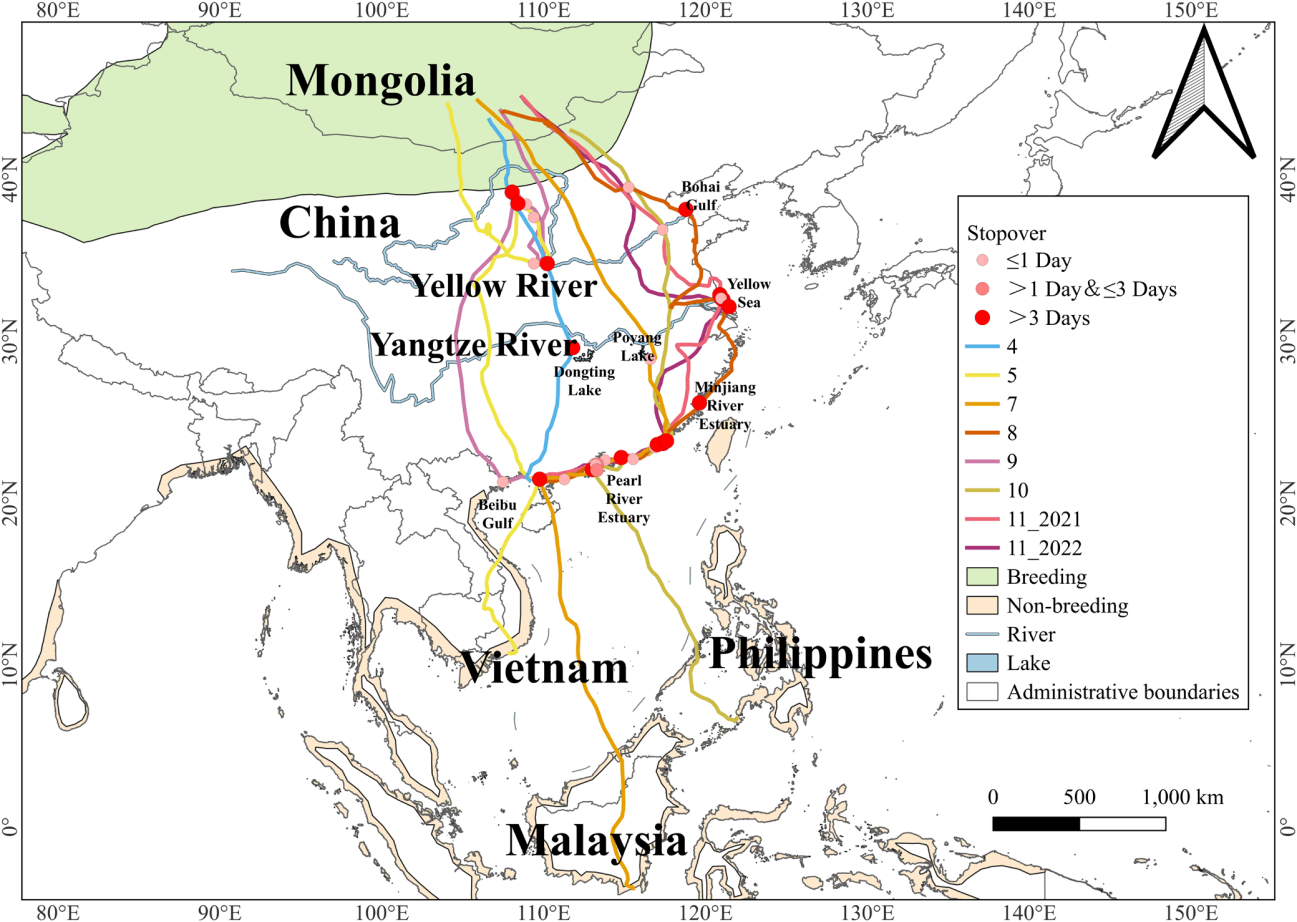
Autumn southward migration routes and stopover sites of GPS‐tracked Greater Sand Plovers from Mongolia to the northern Beibu Gulf and more southerly non‐breeding areas. Lines represent individual migration routes, and circles indicate stopover sites, with darker red symbols representing longer stopover duration. The shaded green and orange areas represent the breeding and non‐breeding ranges, respectively, and major rivers, lakes, and coastal reference sites are shown to aid geographic interpretation.

We recorded eight autumn migration routes from seven tracked individuals during southward migration during 2021–2022, because one individual contributed autumn migration data from 2 years (Figure 3). Autumn migration spanned late June to September, with a mean duration of 33.53 ± 15.97 days (Table S2). Departures occurred between 25 June and 22 July, and arrivals in the Beibu Gulf took place between 8 July and 7 September. A subset of birds continued beyond Beibu to more southerly wintering grounds (*n* = 3), arriving on 21 August, 25 August, and 10 September, whereas the remainder appeared to remain in the Beibu Gulf. Individuals departed from breeding grounds in Mongolia and followed multiple southward migration pathways to reach wintering areas in the Beibu Gulf and far south to Southeast Asia, including Vietnam, Malaysia, and the Philippines. Some individuals (*n* = 3) migrated along inland routes through central China, while others took coastal pathways along the Chinese coastline (*n* = 5).

### 3.2 Stopovers

During the spring migration, we identified 62 stopover sites. On average, each bird used 5.16 ± 2.03 sites and remained at each for 1.63 ± 0.48 days (Table S2). Most stopovers occurred in wetlands along the Yellow River and Yangtze River basins, with additional use of artificial reservoirs. Stopovers lasting more than 3 days were recorded at Dongting Lake and along the Yellow River in China. During the autumn migration, a total of 30 stopover sites were identified. Each individual utilized an average of 3.75 ± 1.75 stopover sites and stayed for 5.79 ± 1.64 days per site (Table S2). Long stopovers (> 3 days) were recorded along the main course of the Yellow River and in coastal wetlands in China.

### 3.3 Seasonal Differences in Migration Metrics Within the Mongolia–China Segment

**FIGURE 4:**
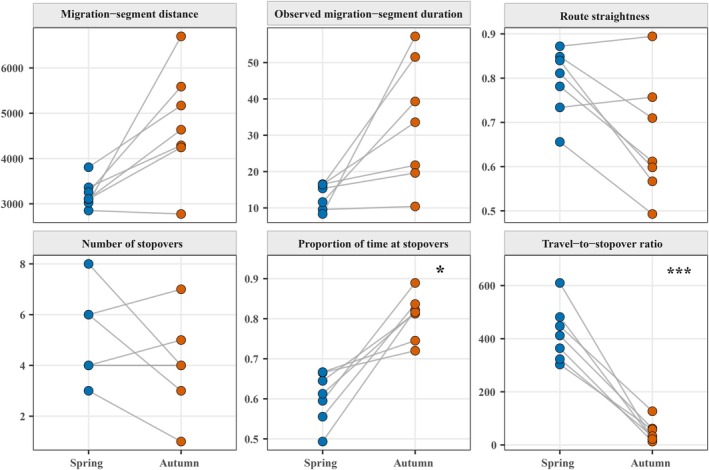
Paired seasonal comparisons of six primary migration metrics in Greater Sand Plovers tracked from the Guangxi coast of the northern Beibu Gulf during 2021–2022. Comparisons are based on seven individuals with paired spring and autumn records, with one record retained per bird. Spring refers to northbound migration from the Beibu Gulf region toward Mongolia, and autumn refers to southbound migration from Mongolia toward the Beibu Gulf region. Blue points represent spring values, orange points represent autumn values, and gray lines connect paired seasonal values from the same individual. Metric definitions are provided in Table 2. *p*‐values were adjusted across the six primary metrics using the Holm‐Bonferroni method. Significant seasonal differences after correction were detected for proportion of time at stopovers (*Holm‐adjusted *p* = 0.013) and travel‐to‐stopover ratio (***Holm‐adjusted *p* < 0.001); the other four metrics were not significant after correction.

Seasonal comparisons were conducted for seven individuals with paired spring and autumn records, with one record retained per bird. Across the six primary migration metrics, Greater Sand Plovers showed consistent directional seasonal differences in several aspects of migration, although only two metrics remained significant after Holm‐Bonferroni correction (Figure 4).

Autumn migration segments were longer in distance and duration than spring migration segments. Migration‐segment distance averaged 3217.39 ± 307.33 km in spring and 4771.87 ± 1227.80 km in autumn, but this difference did not remain significant after correction for multiple comparisons (paired *t*‐test, *t* = 3.485, df = 6, raw *p* = 0.013, Holm‐adjusted *p* = 0.052). Observed migration‐segment duration also tended to be longer in autumn than in spring (spring: 13.45 ± 3.52 days; autumn: 33.35 ± 17.25 days; paired *t*‐test, *t* = 2.905, df = 6, raw *p* = 0.027, Holm‐adjusted *p* = 0.068). Route straightness was lower in autumn than in spring, indicating a directional tendency toward less direct autumn routes, but this difference was not significant after correction (spring: 0.79 ± 0.08; autumn: 0.66 ± 0.14; paired *t*‐test, *t* = −3.046, df = 6, raw *p* = 0.023, Holm‐adjusted *p* = 0.068). The number of stopovers did not differ significantly between seasons (spring: 5.00 ± 1.73; autumn: 4.00 ± 1.83; paired *t*‐test, *t* = −1.323, df = 6, raw *p* = 0.234, Holm‐adjusted *p* = 0.234).

The strongest seasonal differences were associated with stopover allocation and travel relative to stopover investment. The proportion of time spent at stopovers was significantly higher in autumn than in spring (spring: 0.60 ± 0.06; autumn: 0.81 ± 0.06; paired *t*‐test, *t* = 4.968, df = 6, raw *p* = 0.003, Holm‐adjusted *p* = 0.013). By contrast, the travel‐to‐stopover ratio was significantly higher in spring than in autumn (spring: 420.15 ± 105.61 km/day; autumn: 47.92 ± 39.58 km/day; paired *t*‐test, *t* = −8.461, df = 6, raw *p* < 0.001, Holm‐adjusted *p* < 0.001). Together, these results indicate that spring movements involved greater progression per unit stopover time, whereas autumn movements were characterized by greater allocation of migration time to stopovers.

## 4 Discussion

### 4.1 Seasonal Differences in Migration and Stopover Use Within the Mongolia–China Segment

Our results are broadly consistent with previously described migration routes and habitat associations of Greater Sand Plovers (BirdLife International 2019; Wetlands International 2025). By using GPS‐GSM tracking, we characterized spring and autumn movements and assessed seasonal differences in route use between the Guangxi coast of the northern Beibu Gulf, China, and Mongolia. Our data suggest a consistent pattern: during northward migration from the Beibu Gulf to the Mongolian Plateau, tracked individuals generally followed a more inland corridor across China to reach their breeding grounds. In contrast, during southward migration from the Mongolian Plateau, movements more often shifted toward the coastline. Although route‐level differences should be interpreted cautiously because they did not remain significant after correction for multiple comparisons, this seasonal route differentiation resembles patterns reported in other migratory shorebirds. Within the EAAF, comparative tracking of six long‐distance migratory shorebird species showed that prebreeding migration was generally faster and involved fewer stopover sites than postbreeding migration (Zhao et al. 2017). Satellite tracking of Whimbrels showed seasonal and population differences in migration routes and stopover‐region use, including differential use of the Yellow Sea, coastal China, Japan, and more southerly sites during northward and southward migration (Kuang et al. 2020). Similar seasonal contrasts have also been reported in other flyways, such as Gray Plovers in Europe, where spring movements were narrower and more direct than autumn movements (Exo et al. 2019). Predominant wind patterns may also contribute to seasonal differences in shorebird migration routes and timing. For example, recent work on Far Eastern Oystercatchers in the EAAF showed that wind and temperature influenced migration decisions across the annual cycle (Zhao et al. 2026). To explore this possibility in Greater Sand Plovers, we conducted an exploratory wind‐support analysis using ERA5 hourly 10‐m wind data (without flight altitude). Across the seven paired individuals, mean tailwind support was positive in spring but negative in autumn (spring: 1.76 ± 1.03 m/s; autumn: −1.72 ± 0.97 m/s; paired *t*‐test, *t* = −6.65, df = 6, Holm‐adjusted *p* = 0.001). The proportion of flight distance with tailwind support was also higher in spring than in autumn (spring: 0.77 ± 0.13; autumn: 0.25 ± 0.12; paired *t*‐test, *t* = −7.65, df = 6, Holm‐adjusted *p* < 0.001). These results suggest that more favorable wind assistance during northward migration may have contributed to the higher travel‐to‐stopover ratio observed in spring. Although 10‐m wind data may not represent wind conditions at the birds' actual flight altitude, they provide a standardized index of near‐surface wind support across seasons and individuals.

Migration is not a fixed routine repeated twice each year, but an optimization process shaped by changing ecological opportunity, physiological constraint, and seasonal urgency (Alerstam et al. 2003; Nathan et al. 2008). Within the migration segment available for direct comparison, Greater Sand Plovers showed seasonal differences in route use and stopover allocation that were broadly consistent with contrasting seasonal constraints in migratory birds (Alerstam and Lindström 1990; Zhao et al. 2017). Spring movements had a significantly higher travel‐to‐stopover ratio, indicating that birds covered more distance per unit stopover time during northward migration. This result suggested that faster spring migration was achieved primarily through more direct and reduced path length, rather than through a reduction in the number of stopovers. This pattern is consistent with broader evidence that pre‐breeding migration in shorebirds is often more time‐constrained than postbreeding migration (Duijns et al. 2019), probably because earlier arrival may confer reproductive benefits such as improved access to territories and mates (Morrison et al. 2019).

By contrast, post‐breeding southward migration showed a different pattern of temporal allocation. During autumn, Greater Sand Plovers spent a significantly greater proportion of the migration period at stopovers than during spring, indicating a stronger allocation of time to stopover use during southward migration. Thus, autumn migration appeared to advance more slowly not because birds used more stopover sites, but because a larger share of the migration period was spent at stopover sites. This pattern is consistent with greater refueling or recovery demands after breeding, as stopover duration and stopover decisions in migratory shorebirds are often linked to fuel accumulation, body condition, and preparation for subsequent migratory flights (Ma et al. 2013; Duijns et al. 2019). It may also be associated with preparation for or progression through post‐breeding molt. The inland‐to‐coastal shift observed in autumn may also reflect greater reliance on coastal habitats that provide predictable foraging resources and nearby high‐tide roosts. Extensive intertidal flats along China's coast may provide high‐quality prey resources and support large numbers of migratory shorebirds during late summer and autumn (Burger et al. 1977; MacKinnon et al. 2012; Sun et al. 2022). Taken together, these results suggest that seasonal route differences in the tracked sample may reflect stronger time constraints in spring and greater refueling or recovery demands in autumn, potentially associated with molt and post‐breeding condition recovery (Alfaro et al. 2018).

### 4.2 The Northern Beibu Gulf as a Dual‐Function Region Shaping Seasonal Behavior of Greater Sand Plovers Along the EAAF

Although globally assessed as Least Concern, the global population trend of the Greater Sand Plover is declining. At the flyway level, evaluations have considered the EAAF population a candidate for uplisting to Vulnerable since 2008, with this status reaffirmed in recent reviews (IUCN 2023). These evaluations suggest that the species may face a higher level of conservation concern than indicated by its current global category, especially along the EAAF.

**FIGURE 5:**
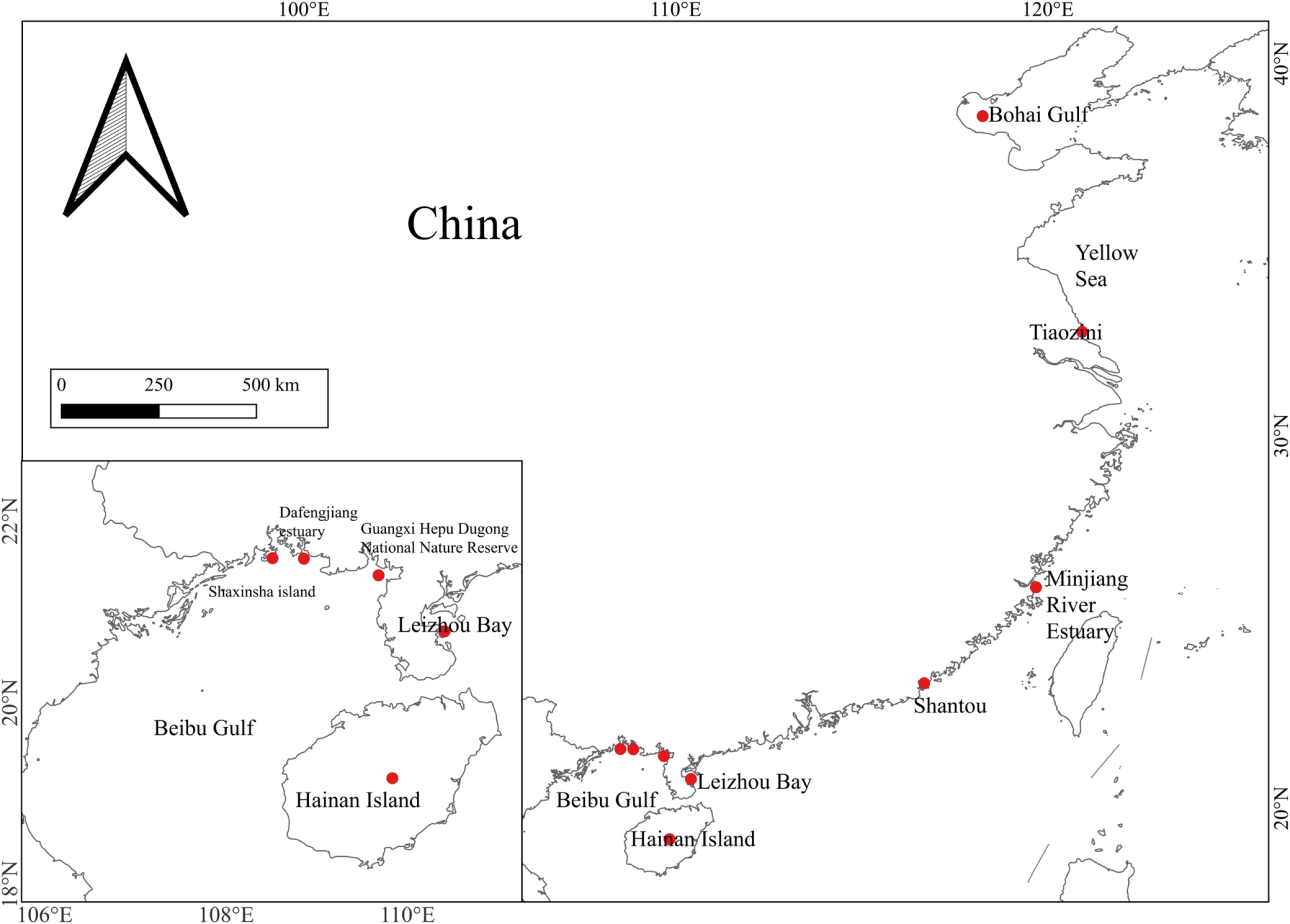
Locations of the northern Beibu Gulf and other Chinese coastal sites mentioned in the Discussion. Sites within or adjacent to the northern Beibu Gulf include Hepu Dugong National Nature Reserve, Shankousha Island, Dafengjiang Estuary, and Leizhou Bay. Other coastal reference sites, including Shantou, the Minjiang Estuary, Tiaozini, and Bohai Bay, are located farther east or north along the Chinese coast.

Coastal surveys reveal a pronounced spatial gradient in the non‐breeding distribution of Greater Sand Plovers along the Chinese coast (Figure 5). In Guangdong, more than 1000 wintering individuals were recorded at Shantou and Leizhou Bay (Min et al. 2020). On Hainan Island, the maximum winter count was just over 800 individuals across the island (Fei et al. 2021). Numbers were substantially higher in the Beibu Gulf (Guangxi), where over 4000 Greater Sand Plovers were recorded in the Hepu Dugong National Nature Reserve during both migration and wintering periods (Tan et al. 2025), with approximately 2500 at the Dafengjiang estuary (Unpublished results), and around 1500 on Shanxinsha Island (Liu et al. 2023). These consistently high counts across both migratory and non‐breeding periods suggest that the northern Beibu Gulf does not act merely as a transient stopover area, but also functions as a major northern non‐breeding region for the species along the EAAF. In contrast, well‐known coastal wetlands further north and east, such as Bohai Gulf, Tiaozini in the Yellow Sea and the Minjiang estuary in Fujian (Choi et al. 2020), are used predominantly as stopover sites (Lin et al. 2024). Taken together, these patterns indicate that many individuals terminate their southward migration in the Beibu Gulf rather than dispersing more evenly across other coastal wetlands at similar latitudes.

Evidence from this study, local survey counts, and resightings of marked birds together support the interpretation of the Beibu Gulf as both a non‐breeding and migratory stopover region for Greater Sand Plovers. In this study, one tracked individual (ID 11) remained in the Beibu Gulf during the non‐breeding period, suggesting that the region can serve as a non‐breeding endpoint for at least part of the tracked population. This interpretation is consistent with high winter counts reported from local surveys in the Beibu Gulf region (Tan et al. 2025). In addition, several tracked individuals from this study (IDs 4, 5, and 10) used the Beibu Gulf briefly during spring before continuing northward, indicating that the region also functions as a stopover site during northward migration. Resightings of Australian‐ringed or flagged Greater Sand Plovers in Beibu Gulf wetlands during migration further indicate connectivity between the Beibu Gulf and more southerly non‐breeding areas (Yi et al. 2023).

Although based on a small sample, this convergence‐then‐divergence pattern is consistent with movement patterns described in other EAAF shorebirds. For example, Bar‐tailed Godwits and Great Knots from non‐breeding areas in north‐western Australia use the Yellow Sea as a major stopover region before continuing to breeding areas in the Russian Arctic, and Bar‐tailed Godwit subspecies tracked from Australasia similarly converge on East Asian stopover areas before dispersing to different breeding regions (Gill et al. 2009; Battley et al. 2012; Chan et al. 2023). Similar dual seasonal functions have also been reported for Western Sandpipers in San Francisco Bay, where the estuary supports both non‐breeding individuals and northward migrants using the site as a stopover region (Warnock et al. 2026). These examples support the idea that dual‐function coastal regions can shape migration behavior beyond their role as simple passage sites. In our study, the Beibu Gulf appeared to play an analogous, although more southerly, role: tracked Greater Sand Plovers converged there during autumn, after which some remained in the region whereas others continued farther south (Hedenström and Alerstam 1997; Gill et al. 2009; Winger and Pegan 2021; Wen et al. 2024; You et al. 2024; Thevarajan et al. 2025).

These patterns of repeated use of the Beibu Gulf are consistent with our analysis of seasonal movement and stopover allocation. In autumn, the greater proportion of time spent at stopovers suggests more extensive refueling and post‐breeding recovery. This may also be relevant to molt, because adult Greater Sand Plovers are reported to begin primary molt soon after arrival on the non‐breeding grounds, with molt duration varying among non‐breeding regions (Jackson 2018). This reflects an energy and risk‐minimizing strategy, which is naturally facilitated by coastal refueling systems such as Beibu's intertidal flats. Crucially, because Beibu Gulf functions as both non‐breeding endpoint and corridor pinch‐point, habitat quality in this region may simultaneously influence migratory refueling performance and non‐breeding survival or body‐condition maintenance. Consequently, degradation of intertidal flats or loss of roost availability could affect not only immediate migration success but also carry over effects across subsequent stages of the annual cycle. Moreover, local habitat degradation (e.g., loss of high‐tide roost networks, prolonged disruption of tidal access) could generate nonlinear, flyway‐scale impacts (Liu et al. 2022). We therefore recommend recognizing Beibu Gulf explicitly as a dual‐function site in regional and international coordination and maintaining predictable intertidal exposure, foraging and roosting cycles through seasonal management measures, such as disturbance‐free buffer zones around key high‐tide roosts and intertidal foraging areas during peak migration and non‐breeding periods.

### 4.3 Limitations and Future Directions

Several limitations should be acknowledged. First, because all tags were deployed in the Beibu Gulf, spring records are left‐truncated for birds that staged there after moving north from more southerly non‐breeding areas. Second, the number of complete annual and complete seasonal tracks was limited, reducing our ability to generalize seasonal strategies at the population level. Third, tracked individuals did not all follow the same annual pattern: some appeared to terminate autumn migration in the Beibu Gulf, whereas others continued farther south. This individual variation is important because shorebirds can show substantial flexibility in route choice, timing, stopover use, and non‐breeding destination. Consequently, conservation planning for Greater Sand Plovers should not assume a single fixed migration tactic, but should account for alternative annual patterns and the network of sites that support them. Fourth, most deployments did not span more than a single annual cycle, potentially because of transmitter failure, harness loss, mortality, reduced solar charging, or GSM interruption, which constrained assessment of interannual repeatability. To reduce these uncertainties, future work should focus on deploying more advanced, longer lasting tags and expanding marking across multiple sites and seasons along the flyway, thereby increasing sample sizes and capturing a broader spectrum of routes and tactics at the flyway scale.

## 5 Conclusion

Migratory shorebirds with different travel distances and life‐history traits are likely to respond unevenly to climate and land‐use change. Therefore, proactive protection is essential for maintaining healthy populations, which underscores the need to understand species‐specific migration routes and movement behavior (Cooke et al. 2023; Cardillo et al. 2026). In our study, autumn movements of Greater Sand Plovers involved a greater proportion of time at stopovers, whereas spring movements showed a higher travel‐to‐stopover ratio. Route‐level metrics showed directional seasonal patterns but were not significant after correction for multiple comparisons and therefore require confirmation with larger samples. Across tracked individuals, the northern Beibu Gulf functioned as a dual‐purpose region, serving both as a non‐breeding area and as a migratory stopover region. Protecting the northern Beibu Gulf, including intertidal foraging areas, high‐tide roosts, and connected inland and coastal wetlands used by Greater Sand Plovers, will be important for conserving this species and other migratory shorebirds along the EAAF.

## Author Contributions

**Lei Xu:** conceptualization (equal), data curation (equal), formal analysis (equal), methodology (equal), visualization (equal), writing – original draft (equal), writing – review and editing (equal). **Zhiying Cheng:** data curation (equal), investigation (equal), resources (equal). **Tao Meng:** investigation (equal), resources (equal). **Gang Yang:** data curation (equal), funding acquisition (equal), investigation (equal), project administration (equal), resources (equal), supervision (equal). **Aiwu Jiang:** conceptualization (equal), funding acquisition (equal), supervision (equal), writing – review and editing (equal).

## Funding

This project was supported by Data Processing and Report Compilation of Migratory Bird Routes in Guangxi funded by Guangxi Wildlife Conservation Association (ST2022559), Guangxi Science and Technology Base and Talent Special Project (AD25069066), Guangxi Migratory Bird Flyway and Stopover Site Survey Program and Wild Animal and Plant Artificial Breeding (Cultivation) Monitoring Program funded by Guangxi Forestry Bureau (GXZC2020‐C3‐002380‐GXKW).

## Ethics Statement

All procedures involving the capture, handling, and tagging of Greater Sand Plovers were carried out in accordance with the relevant guidelines and regulations for the use of wild birds in research and with the laws of the People's Republic of China. Fieldwork was conducted under permits issued by the Forestry Administration of Guangxi Zhuang Autonomous Region and the administrative authorities.

## Conflicts of Interest

The authors declare no conflicts of interest.

## Supporting information


**Table S1:** Tracking summary and data completeness for 18 GPS–GSM tagged. Deployment outcomes were assessed from terminal tracking data. We did not conduct systematic carcass searches or necropsies; therefore, causes of transmitter loss, signal cessation, or apparent mortality could not be determined. Individuals were retained for route description only when they provided identifiable spring and/or autumn migration segments. Paired seasonal comparisons were restricted to individuals with both spring and autumn records, with one record retained per bird.
**Table S2:** Spring and autumn migration schedules and movement metrics of GPS‐tracked Greater Sand Plovers along the EAAF.

## Data Availability

The data supporting the results of this study have been deposited in Dryad. The reserved permanent Dryad DOI for this dataset is https://doi.org/10.5061/dryad.8gtht7749.
