# Supplementary material for: Seasonal Differences in Migration Routes and Stopover Use of Greater Sand Plovers Between Mongolia and the Beibu Gulf Revealed by GPS Tracking
Source: Ecol Evol. 2026 Jun 29;16(7):e73914. doi: 10.1002/ece3.73914 (PMC13314145; doi:10.1002/ece3.73914)
Supplement: Supplementary file 1 — Table S1: Tracking summary and data completeness for 18 GPS–GSM tagged. Deployment outcomes were assessed from terminal tracking data. We did not conduct systematic carcass searches or necropsies; therefore, causes of transmitter loss, signal cessation, or apparent mortality could not be determined. Individuals were retained for route description only when they provided identifiable spring and/or autumn migration segments. Paired seasonal comparisons were restricted to individuals with both spring and autumn records, with one record retained per bird. Table S2: Spring and autumn migration schedules and movement metrics of GPS‐tracked Greater Sand Plovers along the EAAF. [file ECE3-16-e73914-s001.docx]

**Table S1** Tracking summary and data completeness for 18 GPS–GSM tagged. Deployment outcomes were assessed from terminal tracking data. We did not conduct systematic carcass searches or necropsies; therefore, causes of transmitter loss, signal cessation, or apparent mortality could not be determined. Individuals were retained for route description only when they provided identifiable spring and/or autumn migration segments. Paired seasonal comparisons were restricted to individuals with both spring and autumn records, with one record retained per bird.

| **Bird ID** | **Capture site** | **Deployment date** | **Last signal date** | **Included in route description (Y/N)** | **Included in paired seasonal comparison (Y/N)** | **Deployment outcome based on terminal data** | **Reason for exclusion or limitation** |
| --- | --- | --- | --- | --- | --- | --- | --- |
| 1 | BHCP | 2021-04-19 | 2021-05-15 | Y | N | Provided identifiable spring segment only | Retained for route description but not paired comparison |
| 2 | BHCP | 2021-03-28 | 2021-05-05 | Y | N | Provided identifiable spring segment only | Retained for route description but not paired comparison |
| 3 | HPDR | 2021-03-31 | 2021-07-11 | Y | N | Provided identifiable spring segment only | Retained for route description but not paired comparison |
| 4 | BHCP | 2021-04-19 | 2021-09-12 | Y | Y | Provided identifiable migration segment(s) | Retained for analysis |
| 5 | HPDR | 2021-04-19 | 2021-09-23 | Y | Y | Provided identifiable migration segment(s) | Retained for analysis |
| 6 | SLL | 2021-03-17 | 2021-08-31 | Y | N | Provided identifiable spring segment only | Retained for route description but not paired comparison |
| 7 | HPDR | 2021-03-29 | 2021-09-14 | Y | Y | Provided identifiable migration segment(s) | Retained for analysis |
| 8 | SLL | 2021-03-17 | 2021-10-26 | Y | Y | Provided identifiable migration segment(s) | Retained for analysis |
| 9 | HPDR | 2021-04-19 | 2021-12-02 | Y | Y | Provided identifiable migration segment(s) | Retained for analysis |
| 10 | BHCP | 2021-04-19 | 2022-03-26 | Y | Y | Provided identifiable migration segment(s) | Retained for analysis |
| 11 | HPDR | 2021-03-31 | 2022-10-24 | Y | Y | Provided identifiable migration segment(s) | Only one annual record retained in paired analysis to avoid pseudoreplication |
| 12 | SLL | 2021-03-17 | 2021-05-13 | N | N | No identifiable migration segment for this analysis based on terminal inspection | Excluded from migration analyses |
| 13 | SLL | 2021-03-17 | 2021-03-21 | N | N | Transmission ended shortly after deployment; final outcome inferred from terminal data | Excluded because no usable migration data were obtained |
| 14 | SLL | 2021-03-31 | 2021-05-10 | N | N | No identifiable migration segment for this analysis based on terminal inspection | Excluded from migration analyses |
| 15 | HPDR | 2021-03-31 | 2021-05-08 | N | N | Insufficient or ambiguous movement data for defining migration segment | Excluded from migration analyses |
| 16 | HPDR | 2021-03-31 | 2021-07-14 | N | N | Insufficient or ambiguous movement data for defining migration segment | Excluded from migration analyses |
| 17 | HPDR | 2021-04-01 | 2021-05-08 | N | N | Insufficient or ambiguous movement data for defining migration segment | Excluded from migration analyses |
| 18 | BHCP | 2021-04-18 | 2021-06-03 | N | N | Insufficient or ambiguous movement data for defining migration segment | Excluded from migration analyses |

**Table S2** Spring and autumn migration schedules and movement metrics of GPS-tracked Greater Sand Plovers along the EAAF.

| ID | Season | Departure date  and site | Arrival date  and site | Total Observed migration-segment duration  (day) | Migration-segment distance (km) | Movement-phase travel rate (km/day) | Number of stopover | Total stopover duration (days) |
| --- | --- | --- | --- | --- | --- | --- | --- | --- |
| 1 | Spring | 2021/4/15 Beibu Gulf | 2021/5/7 Mongolian | 21.44 | 4220.02 | 485.49 | 9 | 12.75 |
| 2 | Spring | 2021/4/16 Beibu Gulf | 2021/4/23 Mongolian | 6.48 | 3011.83 | 897.38 | 3 | 3.12 |
| 3 | Spring | 2021/4/25 Beibu Gulf | 2021/5/10 Mongolian | 14.49 | 4281.72 | 524.64 | 5 | 6.33 |
| 4 | Spring | 2021/5/3 Beibu Gulf | 2021/5/13 Mongolian | 9.58 | 2852.58 | 889.03 | 3 | 6.37 |
|  | Autumn | 2021/7/6Mongolian | 2021/7/17 Beibu Gulf | 10.41 | 2775.57 | 951.56 | 1 | 7.50 |
| 5 | Spring | 2021/4/20Beibu Gulf | 2021/5/7 Mongolian | 16.55 | 3363.94 | 400.99 | 6 | 8.16 |
|  | Autumn | 2021/7/5 Mongolian | 2021/7/27 Vietnam | 21.74 | 4290.64 | 1107.29 | 3 | 17.87 |
| 6 | Spring | 2021/4/25 Beibu Gulf | 2021/5/10 Mongolian | 15.12 | 2644.31 | 634.59 | 7 | 10.95 |
| 7 | Spring | 2021/4/19 Beibu Gulf | 2021/4/28 Mongolian | 8.33 | 3024.14 | 895.96 | 4 | 4.95 |
|  | Autumn | 2021/6/25 Mongolian | 2021/8/21 Malaysia | 57.20 | 6697.38 | 1057.48 | 4 | 50.87 |
| 8 | Spring | 2021/4/22 Beibu Gulf | 2021/5/9 Mongolian | 16.21 | 3808.30 | 661.08 | 6 | 10.45 |
|  | Autumn | 2021/7/17 Mongolian | 2021/9/7 Beibu Gulf | 51.54 | 5169.96 | 534.84 | 7 | 41.87 |
| 9 | Spring | 2021/4/23 Beibu Gulf | 2021/5/9 Mongolian | 15.37 | 3104.75 | 605.84 | 8 | 10.25 |
|  | Autumn | 2021/7/9 Mongolian | 2021/7/29 Beibu Gulf | 19.62 | 4245.09 | 848.97 | 4 | 14.62 |
| 10 | Spring | 2021/5/13 Beibu Gulf | 2021/5/30 Mongolian | 16.45 | 3257.13 | 510.92 | 4 | 10.08 |
|  | Autumn | 2021/7/22 Mongolian | 2021/8/25 Philippines | 33.58 | 5588.05 | 906.16 | 4 | 27.41 |
| 11 | Spring | 2021/4/23 Beibu Gulf | 2021/5/5 Mongolian | 11.62 | 3110.83 | 601.57 | 4 | 6.45 |
|  | Autumn | 2021/7/9 Mongolian | 2021/8/18 Beibu Gulf | 39.29 | 4636.35 | 722.48 | 5 | 32.87 |
|  | Spring | 2022/4/20 Beibu Gulf | 2022/5/1 Mongolian | 10.70 | 2858.00 | 836.48 | 3 | 7.29 |
|  | Autumn | 2022/7/16 Mongolian | 2022/8/20 Beibu Gulf | 34.87 | 4663.58 | 1055.89 | 4 | 30.45 |
